# Implementing Child-focused Activity Meter Utilization into the Elementary School Classroom Setting Using a Collaborative Community-based Approach

**DOI:** 10.4172/2161-0711.1000379

**Published:** 2015-11-25

**Authors:** BA Lynch, A Jones, BK Biggs, T Kaufman, V Cristiani, S Kumar, S Quigg, J Maxson, L Swenson, N Jacobson

**Affiliations:** 1 Department of Pediatric and Adolescent Medicine, USA; 2 Division of Child and Adolescent Psychology, Department of Psychiatry and Psychology, USA; 3 Department of Family Medicine, USA; 4 Division of Pediatric Endocrinology, Department of Pediatric and Adolescent Medicine, USA; 5 Division of Endocrinology, Department of Medicine, USA; 6 Section of Patient Education, Mayo Clinic, Rochester, MN, USA

**Keywords:** Assistive technology, Body mass index, Childhood obesity, Obesity, Overweight, Pediatric obesity, Physical activity, Schools

## Abstract

**Introduction:**

The prevalence of pediatric obesity has increased over the past 3 decades and is a pressing public health program. New technology advancements that can encourage more physical in children are needed. The Zamzee program is an activity meter linked to a motivational website designed for children 8–14 years of age. The objective of the study was to use a collaborative approach between a medical center, the private sector and local school staff to assess the feasibility of using the Zamzee Program in the school-based setting to improve physical activity levels in children.

**Methods:**

This was a pilot 8-week observational study offered to all children in one fifth grade classroom. Body mass index (BMI), the amount of physical activity by 3-day recall survey, and satisfaction with usability of the Zamzee Program were measured pre- and post-study.

**Results:**

Out of 11 children who enrolled in the study, 7 completed all study activities. In those who completed the study, the median (interquartile range) total activity time by survey increased by 17 (1042) minutes and the BMI percentile change was 0 (8). Both children and their caregivers found the Zamzee Activity Meter (6/7) and website (6/7) “very easy” or “easy” to use.

**Conclusion:**

The Zamzee Program was found to be usable but did not significantly improve physical activity levels or BMI. Collaborative obesity intervention projects involving medical centers, the private sector and local schools are feasible but the effectiveness needs to be evaluated in larger-scale studies.

## Background

The prevalence of pediatric obesity has increased over the past 3 decades and reached 17.7% by 2011–12 [1–3]. The Institute of Medicine recommends making schools a national focus for obesity prevention efforts [4]. An aspect of this recommendation is that children have at least 60 minutes of moderate-to-vigorous physical activity (MVPA) each day, but most children don’t achieve this goal [5–7]. Multiple reviews and meta-analyses have demonstrated that school-based obesity interventions can improve BMI or healthy behaviors [8–15]. Collaboration between academic medical centers and the private sector in implementing school-based obesity interventions might add further promise.

The use of child-focused accelerometers in the classroom setting to promote physical activity has not been either extensively or rigorously evaluated. Pedometer use in children has been shown to increase physical activity levels; however, decreases in BMI have generally not been observed [16]. The Zamzee Program is an activity meter linked to a motivational website designed for children 8–14 years of age [17]. The primary aim of this study was to design a collaborative project between the Mayo Clinic, the Zamzee Company, and the Rochester Public Schools to test the feasibility and usability of utilizing the Zamzee Program in the classroom setting. A secondary aim of this study is to evaluate if child activity levels and BMI change during the seven-week study period.

### Description of Initiative

#### Pre-study planning

This study is a part of a series of community collaborative studies addressing childhood obesity. The prior study was a collaborative obesity initiative in our local public schools [18]. Zamzee Company staff, Mayo Clinic researchers and staff, and Rochester Public School District staff participated in a pre-study webinar meeting where the capabilities of the Zamzee Program were discussed.

The protocol was developed as a result of multiple planning meetings between the school staff, who devised how to complete study procedures within the school setting, and Mayo Clinic researchers and staff, who obtained Institutional Review Board approval and organized the research consent process.

Zamzee provided technical support for this study but did not participate with the project conception or design. The study was reviewed and approved by the Mayo Clinic Institutional Review Board and the Rochester Public School Board.

### Participants

Twenty-nine students in a fifth grade class at a public elementary school in Rochester, MN, and their caregivers were offered the opportunity to participate in this study.

Caregiver was defined as the child’s biological parent, legally adoptive parent or legal guardian. Of these 29, 11 agreed to be in study. Of the 11 enrolled, 1 was excluded from part of the analysis due to lack of appropriate legal guardian consent to obtain data from Zamzee. No students left or joined the class during the study period of April 15, 2014 through June 4, 2014.

### Instruments

#### Zamzee program

The Zamzee Program consists of a 3-axis accelerometer-based activity meter and website, designed to motivate users to increase physical activity. The website is approved for child use with criteria defined by the Child Online Privacy Protection Act [19].

Results from the meter are uploaded to a child-friendly, secure website (password protected) where children and their caregivers can review the child’s physical activity results, complete physical activity challenges, and earn healthy prizes. This device records the minutes of moderate-to-vigorous physical activity as well as “minutes in the zone”, a measure of total physical activity.

A calibration study in 31 middle-school children found that the Zamzee Activity Meter was a valid measure of MVPA with a sensitivity of 85.9%, specificity of 97.5%, and r of 0.94 correspondence with the RT3 [20] accelerometer system [21].

#### Participant surveys

Children and caregivers reported their satisfaction with the use of the child-focused activity meter and associated website pre- and post-study.

Survey questions were developed by investigators with answers recorded on a Likert Scale. The questions assessed usability, perceived value, and perceived effect of the Zamzee Program on the child’s physical activity.

#### Demographic survey

Caregivers completed a demographic survey to report their child’s race/ethnicity, age, sex, and primary insurance type (private vs public vs none) as well as their relationship to the child, marital status, education level, employment status, age, and race/ethnicity.

#### The self-administered physical activity checklist (SAPAC) survey

The SAPAC is a reliable and valid instrument for measuring exercise levels in elementary school age children [22]. As part of this measure, children reported time engaged in a list of specific physical activities and screen time over the preceding 3 days.

## Procedure

This was an observational study comparing children pre- and post-intervention. Families were invited to participate in the study by a mailed contact letter with accompanying consent form. Families who decided to participate were invited to a pre-study meeting at the participating school where the study was discussed in detail using PowerPoint slides.

In addition, a light meal, interpreter services, and childcare for younger siblings was provided. Mayo Clinic research staff then reviewed the written parental/caregiver consent and participant (child) assent forms individually with each family in a private setting.

Potential enrollees were given the option to sign at that time, to decline participation, or to consider further (study coordinator contact information was provided). After study enrollment, the SAPAC activity survey and a pre-study survey related to the activity meter and website were completed by students.

Participating caregivers completed a pre-study survey as well as a family demographic survey. Mayo Clinic staff measured the child’s height and weight (for BMI calculation [23]) in a private room, while wearing light clothing.

As a last step to the pre-study meeting, Mayo Clinic staff distributed activity meters to the children and assisted caregivers with the registration of their child on the Zamzee website. As a part of this registration, caregivers agreed to the terms and conditions created by Zamzee which discussed guidelines for website use and procedures to protect the child’s data.

Following study enrollment, Zamzee emailed each participating caregiver a consent form that sought authorization for Zamzee to share the website data results with Mayo Clinic researchers.

There were 3 “competition” periods occurring every 3 weeks (Thursday to Sunday) during which the classroom teacher encouraged participating students to wear the activity meters. Competition periods occurred during the first, fourth, and seventh weeks of the study.

Children could use the Zamzee Program at other times during the month, but no reminders were utilized at those times. Children could compare their results to the other children in the class and could post fun messages (from a child-appropriate list) on the website using a de-identified codename.

Any lost or damaged activity meters were replaced at no cost to the participant during the study period.

At the end of the study, there was a post-study evening meeting at the school where a small meal was provided and childcare and interpretative services were once again available.

Families were reminded of the meeting both by letter from Mayo Clinic researchers and by the regular electronic communication from the classroom teacher. The SAPAC survey was completed by students and the post-study satisfaction surveys were completed by both the participating students and their caregiver.

The child’s height and weight were measured and BMI was calculated by study team members in the same manner as pre-study.

Using mailed letters from Mayo Clinic researchers and electronic communication from the classroom teacher, families who couldn’t attend the post-study evening meeting were offered the opportunity to meet at the school with Mayo Clinic researchers to complete the post-study activities at another accommodating time.

### Data analysis

The Mayo Clinic research staff had access to the Zamzee Program Leader Dashboard, where data from the activity meters could be reviewed.

BMI data and all survey (demographic, SAPAC, child and parent satisfaction) data were recorded using a keycode in REDCAP [24] and then exported to Excel for the analysis. The median and interquartile range (IQR) values were determined for: 1) SAPAC total activity minutes pre and post study; 2) BMI percentile pre and post study; 3) minutes of physical activity.

## Results

Of the 11 students enrolled in the study, 7 completed all study activities. Out of the 11 students enrolled in our study, 73% were female, 55% were white and 50% had private insurance. The caregivers of these students were all greater than age 30 years, with 64% married, 73% having at least some college education and 64% employed full-time.

Overall, survey data found that the Zamzee Program was usable. On the pre-study survey, 86% of caregivers whose child completed the study did something to get their child active either daily or weekly.

On the post-study survey, both students and caregivers found the Zamzee (86%) and website (86%) were “very easy” or “easy” to use. All students who completed the post-study survey felt that the Zamzee Program helped them become more active; 57% indicated the website helped them become more active “a little” and 43% felt the website helped “a lot”. Of caregivers completing the post-study survey, 86% indicated that both the Zamzee Program and its associated website helped either “a little” or “a lot” to make their child be more active.

Post-study survey results also indicated that all caregivers felt that their child would continue to use the Zamzee Program after the study. During the 7 week study, 2 activity meters were lost and 1 was broken.

Baseline median (Interquartile range [IQR]) 3-day total physical activity time (measured by the SAPAC survey) was 420 (225) minutes and BMI percentile was 60 (65).

In those children who completed the study, the median (IQR) total activity time by survey increased by 17 (1042) minutes (not statistically significant) and the median (IQR) BMI percentile change was 0 (8).

**Figure 1:**
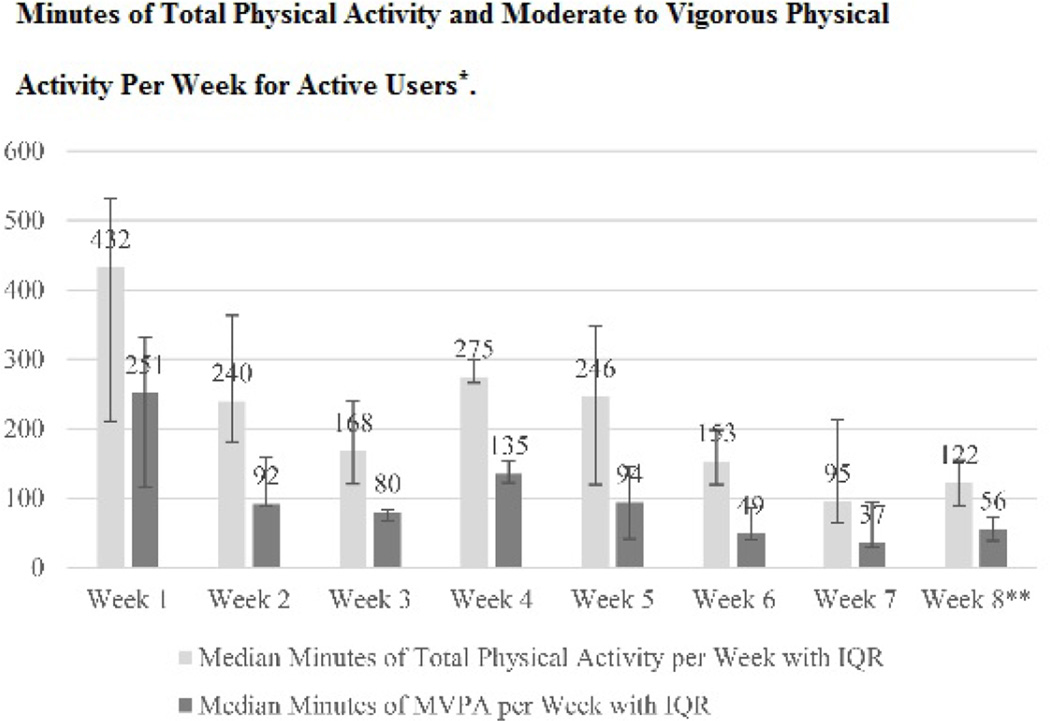
Participant Physical Activity Levels. *Minutes of total physical activity and moderate to vigorous physical activity per week for active users. Abbreviations: IQR, Interquartile Range; MVPA, Moderate to Vigorous Physical Activity; *Active users are participants who uploaded any data during the reporting week; **Week 8 includes 3 total days.

Children’s weekly physical activity level (Figure 1) peaked during the first week and had a second lower peak during the 4th week of the study.

**Figure 2:**
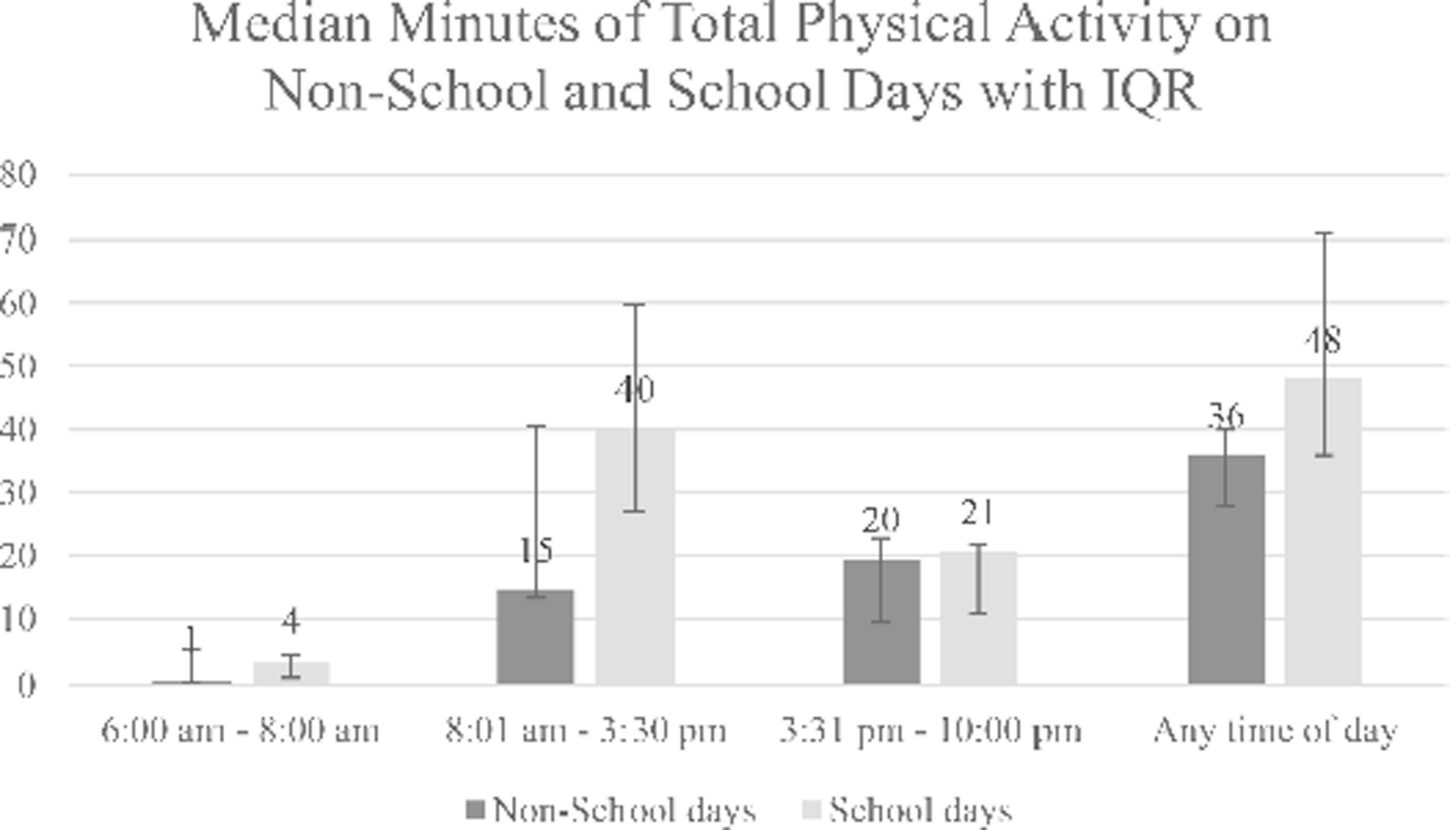
Impact of physical environment and time of day on physical activity levels. Median minutes of total physical activity on non-school and school days with IQR. Abbreviation: IQR: Interquartile Range.

The impact of physical environment (school versus non-school days) on total physical activity is described in Figure 2. The median (IQR) minutes of total activity were 48 (25) minutes on school days and 36 (12) minutes on non-school days. Most of this difference was noted during the school day period where the median (IQR) activity minutes were 40 (33) minutes on school days and 15 (27) minutes on non-school days.

## Discussion

This pilot study demonstrates the feasibility of a healthy lifestyle intervention project using an activity meter in the elementary school setting. The study found that a majority of both caregivers and children who utilized the activity meter were satisfied with the usability of the Zamzee Program.

After obtaining proper consent from participating children and their parents, individual-level Zamzee Program data could be shared with study investigators as well as participants. This model of data sharing offers the opportunity for clinicians or researchers to remotely monitor children’s activity level. This could innovate how clinicians monitor activity levels and provide a new intervention tool to combat pediatric obesity. Alternatively, real-time access to activity level data through programs like Zamzee can aid children and their parents in understanding and modifying the child’s true activity level as parents typically underestimate children’s activity levels. [25] Communication between study investigators, the Zamzee Company, and study participants allowed efficient replacement and reprogramming of Zamzee meters when they were broken or lost without loss of data. This element allowed our study team to minimize the activity data lost during these time periods.

This is the first study evaluating the use of an activity meter with a child-focused interactive website in the elementary school setting. The results of our study did not show decreases in BMI percentile or significant increases in physical activity time. There is one published, randomized, controlled multi-site Zamzee Program trial of 182 middle school-aged children over 6 weeks [21]. The results of this study showed that the intervention group had 15.26 minutes average daily minutes of moderate to vigorous activity which was 54% greater than a passive control group (p<0.0001) and 68% greater than an active control group that had access to an active videogame (p<0.0001) [21]. Our study was similar to this study in that external motivation provided by teachers and parents was not promoted to aid in activity meter use. It is possible that elementary school age children need more adult reinforcement than middle school age children to successfully utilize activity meters. In contrast to this study, our study compared activity levels pre and post intervention and didn’t have a control group. Evaluating use of child-focused activity meters in the extracurricular class or club setting, where an adult leader can issue challenges and interact directly with participating children, would likely improve engagement rate and may also improve the efficacy of the intervention [26,27].

Our study found increased levels of physical activity during the time periods when children were in school as compared to the same time period on non-school days. A previous meta-analysis found that children accumulated more minutes of moderate to vigorous physical activity during school hours than outside of school time, although the difference was only 2 minutes per day and may not be clinically significant [27]. The school day may offer the opportunity for children to engage in higher intensity activity for specific time periods, but the predominant requirement to be seated during class time may lower average activity intensity during the whole school day [27]. Our results highlight the importance of encouraging physical activity within the school setting.

Our study enrollment and completion rates were suboptimal and one lesson learned from the study was to elicit the reasons for non-participation using surveys or interviews. It is plausible that the use of mailed consent and study description forms rather than direct engagement by the school teacher/staff was not an effective way to inform and recruit families. The pre-study meeting was only offered on one night and this was perhaps a barrier for families due to other schedule conflicts. The added incentives of study participation, including a light meal at the pre- and post-study meetings as well as keeping the activity meter after the study, may not have been robust enough to motivate families to enroll or complete the study. There also may have been caregiver apprehension regarding participation in a school-based research project involving an academic center and a private company.

In conclusion, our study found that the physical activity data can be shared effectively between children and their parents as well as clinicians/investigators through a private company after the appropriate consent of participants. Our study also showed that children and their parents can be satisfied with the usability of the Zamzee Program. Our study did not show significant changes in physical activity level or BMI and future larger-scale studies with longer duration are needed to evaluate the effectiveness of this approach.

## Abbreviations

BMI: Body Mass Index
IQR: Interquartile Range
MVPA: Moderate to Vigorous Physical Activity
SAPAC: Self-Administered Physical Activity Checklist
